# Microbiota-Related Changes in Unconjugated Fecal Bile Acids Are Associated With Naturally Occurring, Insulin-Dependent Diabetes Mellitus in Dogs

**DOI:** 10.3389/fvets.2019.00199

**Published:** 2019-06-27

**Authors:** Albert E. Jergens, Blake C. Guard, Alana Redfern, Giacomo Rossi, Jonathan P. Mochel, Rachel Pilla, Lawrance Chandra, Yeon-Jung Seo, Joerg M. Steiner, Jonathan Lidbury, Karin Allenspach, Jan Suchodolski

**Affiliations:** ^1^Department of Veterinary Clinical Sciences, College of Veterinary Medicine, Iowa State University, Ames, IA, United States; ^2^Gastrointestinal Laboratory, Department of Small Animal Clinical Sciences, College of Veterinary Medicine and Biomedical Sciences, Texas A&M University, College Station, TX, United States; ^3^School of Biosciences and Veterinary Medicine, University of Camerino, Macerata, Italy; ^4^Department of Biomedical Sciences, College of Veterinary Medicine, Iowa State University, Ames, IA, United States

**Keywords:** bile acids, diabetes mellitus, microbiota, lipopolysaccharide, dog, T2DM

## Abstract

Diabetes mellitus (DM) in humans has recently been associated with altered intestinal microbiota. The consequences of intestinal dysbiosis, such as increased intestinal permeability and altered microbial metabolites, are suspected to contribute to the host inflammatory state and peripheral insulin resistance. Human diabetics have been shown to have changes in bile acid (BA) metabolism which may be detrimental to glycemic control. The purpose of this study was to examine BA metabolism in dogs with naturally-occurring, insulin-dependent DM and to relate these findings to changes in the intestinal microbiota. A prospective observational study of adult dogs with a clinical diagnosis of DM (*n* = 10) and healthy controls (HC, *n* = 10) was performed. The fecal microbiota were analyzed by 16S rRNA gene next-generation (Illumina) sequencing. Concentrations of fecal unconjugated BA (fUBA) were measured using gas chromatography and mass spectrometry. Analysis of bacterial communities showed no significant difference for any of the alpha-diversity measures between DM vs. HC dogs. Principal coordinate analysis based on unweighted Unifrac distance metric failed to show significant clustering between dog groups (ANOSIM_Unweighted_: *R* = 0.084; *p* = 0.114). However, linear discriminate analysis effects size (LEfSe) detected differentially abundant bacterial taxa (α = 0.01, LDA score >2.0) on various phylogenetic levels. While *Enterobacteriaceae* was overrepresented in dogs with DM, the proportions of Erysipelotrichia, *Mogibacteriaceae*, and *Anaeroplasmataceae* were increased in HC dogs. Dogs with DM had increased concentration of total primary fUBA compared to HC dogs (*p* = 0.028). The concentrations of cholic acid and the cholic acid percentage of the total fUBA were increased (*p* = 0.028 and *p* = 0.035, respectively) in the feces of DM dogs relative to HC dogs. The levels of lithocholic acid (both absolute value and percentage of the total fUBA) were decreased (*p* = 0.043 and *p* < 0.01, respectively) in DM dogs vs. HC dogs. Results indicate that dogs with DM have both intestinal dysbiosis and associated fUBA alterations. The pattern of dysbiosis and altered BA composition is similar to that seen in humans with Type 2 DM. The dog represents a novel large animal model for advancing translational medicine research efforts (e.g., investigating pathogenesis and therapeutics) in DM affecting humans.

## Introduction

The intestinal microbiota is increasingly recognized as a pivotal environmental factor contributing to development of metabolic diseases in humans, including obesity, insulin resistance and type 2 diabetes mellitus (T2DM) (1–3). Microbes in the gut play an important role in metabolic disturbances by increasing energy extraction from ingested foods, regulating host metabolism and generating low-grade intestinal inflammation. High throughput 16S rRNA sequencing of the gut microbiota of *ob/ob* mice found that their obesity was associated with significant shifts in relative abundance of select bacterial taxa (e.g., Bacteroidetes decreased while Firmicutes were increased) vs. lean controls (4). These same investigators demonstrated that the proportion of Bacteroidetes in obese humans was decreased as compared to intestinal populations in lean humans (5). However, other studies aimed at evaluating altered gut microbial composition and its association with human diabetes have produced conflicting results (3, 6–8).

The intestinal microbiota also influences metabolism of bile acids (BA). Cholic acid and chenodeoxycholic acid are primary BA synthesized from cholesterol in the liver. Following ingestion of a fat- and protein-rich meal, primary BA travel down the intestines where they are then modified by anaerobic bacteria into different secondary BA, primarily deoxycholic and lithocholic acids. Accumulating evidence demonstrates that BA are important signaling molecules regulating hepatic glucose metabolism via farsenoid X receptor (FXR)-mediated pathways (9–11). Primary BA are also involved in energy metabolism due to their interaction with G protein coupled BA receptor (TGR-5) activation and release of glucagon-like peptide-1 (GLP-1) (10, 12). As compared to non-obese healthy subjects, the fasting serum of obese humans contains decreased primary BA but increased secondary BA concentrations (13). Whether similar microbiota-related changes in the bile acid profile are associated with naturally occurring, insulin-dependent diabetes mellitus (DM) in dogs has not been previously investigated.

With this study, we evaluated the fecal unconjugated bile acid (fUBA) profiles of diabetic vs. healthy control dogs, and hypothesized that diabetic dogs would have perturbations in fecal bile acids similar to those reported in humans with T2DM, including alterations in their intestinal microbiota.

## Materials and Methods

### Ethical Animal Use

The collection and analysis of blood and fecal samples from healthy dogs and dogs with spontaneous DM were previously approved by the Iowa State University Institutional Animal Care and Use Committee. Written informed consent was obtained from all owners of healthy and DM dogs enrolled in this trial (IACUC Log number: 9-14-7859).

### Animals and Enrollment

Dogs with naturally occurring, insulin-dependent DM (*n* = 10) and healthy control dogs (HC, *n* = 10) were enrolled from the hospital population at the ISU Lloyd Veterinary Medical Center (2014–2016). Dogs with DM were diagnosed on the basis of historical polyuria-polydipsia, change in appetite, and weight loss accompanied by supportive laboratory abnormalities including sustained hyperglycemia, hypercholesterolemia, and glucosuria (14). Dogs with diabetes were enrolled if they were >2 years of age and weighed >6 kg, were fasted overnight (12 h minimum) prior to diagnostic sampling, had no discernible other diseases (including bacterial urinary tract infection with results confirmed by urine culture/susceptibility testing) and received no medications (including antibiotics) within 3 weeks of presentation.

Control dogs were between 2 and 9 years of age and judged to be healthy on the basis of history and normal physical examination. Additionally, HC dogs could not have received antibiotics for a period of 6 months prior to diagnostic sampling nor had any other medications administered other than prophylactic flea/tick/heartworm preventatives. All HC dogs were fasted for at least 12 h before samples were obtained.

The majority (*n* = 7) of DM dogs were fed a low-fat weight reducing diet^1^ and three DM dogs were fed commercial maintenance rations. In these instances, dogs with DM were fed low-fat, high-fiber diets to combat obesity and to facilitate insulin regulation (15). Control dogs were fed either a commercial maintenance ration (*n* = 5), commercial elimination diet (*n* = 2), or a low-fat weight reducing diet^1^,^2^ (*n* = 3). None of the dogs had a history of antibiotic administration for at least 6 months prior to sample collection.

### Sample Collection

Blood was collected from both groups of dogs in the mornings by routine venipuncture using the jugular or cephalic veins. Serum was separated quickly within 15 min of collection and archived at −80°C until laboratory analysis. Fecal samples were obtained by digital extraction following phlebotomy in most (17/20) dogs. In some instances, clients brought in fresh fecal samples (contained in a plastic bag) which had been voided naturally during the morning of diagnostic sampling. In these instances, feces were maintained chilled by refrigeration (<4 h) until archived at −80°C for later analysis.

### DNA Isolation and Sequencing of 16S rRNA Genes

Total bacterial DNA was extracted from canine stool samples (10 DM and 10 control dogs) using a MoBio Power soil DNA isolation kit (MoBio Laboratories, USA) following the manufacturer's instructions. DNA concentration and quality in the extracts was determined using a NanoDrop 1000 spectrophotometer. Sequencing of the V4 region of the 16S rRNA gene primers 515F (5′-GTGCCAGCMGCCGCGGTAA-3′) to 806R (5′-GGACTACVSGGGTATCTAAT-3″) was performed using Illumina sequencing at the MR DNA laboratory (www.mrdnalab.com, Shallowater, TX, USA).

The obtained sequences were processed and analyzed using QIIME v 1.9 (16) as previously described (17), and were uploaded to Sequence Read Archive at NCBI with accession number SRP122536. Briefly, sequence data was first demultiplexed, quality filtered using the default settings in QIIME, chimeras were filtered from the sequence set using USEARCH against the 97% clustered representative sequences from the Greengenes v 13.8 database, while remaining sequences were clustered into Operational Taxonomic Units (OTUs) by using an open reference approach in QIIME (18). Prior to downstream analysis, sequences assigned as chloroplast, mitochondria, and low abundance OTUs, containing <0.01% of the total reads in the dataset were removed. All samples were rarefied to 97,980 sequences for even depth of analysis. Alpha diversity measures included Chao1, Shannon diversity, and observed OTUs (observed species). Beta diversity was evaluated with the phylogeny based UniFrac distance metric and visualized using Principal Coordinate Analysis (PCoA) plots. Bray-Curtis dissimilarity was also calculated from the data set.

### Fecal Unconjugated Bile Acids

The fecal unconjugated bile acids (fUBA) quantified were cholic acid (CA), chenodeoxycholic acid (CDCA), lithocholic acid (LCA), deoxycholic acid (DCA), and ursodeoxycholic acid (UDCA). For the identification and quantification of unconjugated bile acids, the protocol was adapted and modified from methods previously described (19, 20). Unconjugated CA, CDCA, LCA, DCA, and UDCA were purchased from a commercial supplier (Sigma-Aldrich, St. Louis, MO). Deuterated internal standards CA-d_4_ and LCA-d_4_ were purchased from CDN Isotopes (Quebec, Canada). Hydrochloric acid (37% American Chemical Society reagent), hexane [for high-performance liquid-chromatography (HPLC)], 1-butanol for HPLC, and derivatization agent (Supelco's^®^ Sylon HTP HMDS + TCMS + Pyridine, 3:1:9 Kit) were used for preparation of trimethylsilyl ether (TMS) and butyl ester bile acid derivatives.

Naturally voided fecal samples were collected from healthy dogs and dogs with DM. Approximately 0.5 g of wet feces was aliquoted into a tube (5 mL, 57 × 15.3 mm, polypropylene, Sarstedt, Nümbrecht, Germany) using a spatula (Smart Spatula, USA Scientific, Ocala, FL). Fecal samples were kept frozen at −80°C and then lyophilized overnight (Labconco FreeZone 2.5 Plus, Kansas City, MO). Samples were then pulverized and aliquoted using a spatula (Smart Spatula, USA Scientific, Ocala, FL) into disposable glass centrifuge tubes (5 mL, Kimble-Chase, Rockwood, TN). Aliquots of 10–15 mg of lyophilized feces were used, and concentrations of bile acids were later back calculated according to the precise weight of each aliquot. A total volume of 200 μL of butanol containing the internal standards CA-d_4_ and LCA-d_4_ was added to each fecal sample. Twenty microliters of HCl were then added for a final volume of 220 μL and vortexed for 30 s. Samples were then capped and incubated at 65°C for 4 h. Next, samples were evaporated under nitrogen gas until dryness at 65°C for ~25 min. Two-hundred microliters of TMS-derivatization agent were then added to the sample and incubated at 65°C for 30 min. Following incubation, samples were again evaporated under nitrogen gas until dryness at 65°C (~25 min). Samples were then resuspended in 200 μL of hexane, vortexed briefly then centrifuged for 10 min at 3,200 rcf. A 100 μL aliquot was transferred to a GC/MS vial insert (250 μL glass with polymer feet, Agilent, Santa Clara, CA) and the vial was capped for further downstream analysis.

Gas chromatography (GC) and mass spectrometry (MS) was used (6890N and 5975 inert Mass Selective Detector, Agilent, Santa Clara, CA). The instrument was equipped with an autosampler (7683 Series, Agilent, Santa Clara, CA). A capillary column (DB-1ms Ultra Inert, Agilent, Santa Clara, CA) was used with the following dimensions: length: 30 m, diameter: 0.250 mm, film: 0.25 μm. A 20:1 split ratio was utilized after a 1 μL sample injection with an inlet temperature of 250°C. After injection, oven temperature was held at 150°C for 1 min, then ramped at 21°C per minute to a final temperature of 276°C then held at that temperature for 21 min. Post-data acquisition, the oven was heated to 325°C for 3 min for post-run column cleaning. Helium was used as the carrier gas at a nominal flow rate of 1 mL/min. Flow varied slightly to maintain a retention time lock of cholestane-d_4_ set to elute at 11.4 min. Mass spectral data was analyzed using ChemStation (Agilent's Enhanced Data Analysis in MSD version D.02.002.275). Use of this assay demonstrating perturbations in fUBA in dogs with chronic enteropathy has been recently reported (21).

### Serum Lipopolysaccharide (LPS) Concentration

An LPS test was performed to quantitate the production of Gram-negative bacterial endotoxin associated with low-grade intestinal inflammation in insulin-dependent states of DM dogs (22, 23). Serum samples from 10 control and 10 DM dogs were analyzed for their concentration of LPS using the LAL Chromogenic Endotoxin Quantification Kit according to the manufacturer's instructions.

### Statistical Analysis

Normality was tested using Shapiro-Wilk for all measurements (or variables) in the fUBA dataset. When the assumption did not hold, the non-parametric Mann-Whitney test was used for comparison of the groups (R software version 3.5.1, R Foundation for Statistical Computing, Vienna, Austria, and JMP 10, SAS software Inc.). A Fisher's Exact Test was used to test for proportions when evaluating confounding factors, such as sex distribution between healthy control (HC) dogs and dogs with DM. A statistical software package (GraphPad Prism version 5.04 for Windows, GraphPad Software, La Jolla California USA, www.graphpad.com) was used for generating graphs.

For sequence data, linear discriminant analysis effect size (LEfSe) was used to elucidate bacterial taxa different between groups. LEfSe was used in the Galaxy workflow framework with the parameters set at α = 0.01, LDA score = 2.0. Mann-Whitney test (JMP Pro 11, SAS software Inc.) were performed and adjusted for multiple comparison using a Benjamini-Hochberg procedure with a false discovery rate (FDR) at each taxonomic level. ANOSIM (analysis of similarity) test within PRIMER 6 software package (PRIMER-E Ltd., Luton, UK) was used to analyze significant differences in microbial communities between both dog groups.

The Mann-Whitney test was performed to compare differences in LPS values between the two dog groups. For all statistical analyses, a *p*-value < 0.05 was considered significant.

## Results

The patient demographic data for the canine cohorts is presented in Table 1. In brief, gender, body weight, and body condition score were comparable for diabetic and control groups. For diabetic dogs, 1 dog was newly diagnosed with DM while the remaining 9 dogs were receiving parenteral insulin and considered to have poorly regulated DM based on persistent clinical signs, results of blood (sustained hyperglycemia) and urinalysis (marked glucosuria) testing. None of the enrolled animals was ketoacidotic based on the absence of ketonuria and metabolic acidosis on laboratory analysis.

**Table 1:** Patient characteristics.

**Group**	**Age (years)**	**Sex**	**Weight (kg)**	**BCS (0–9)**
**A. PATIENT DEMOGRAPHICS**				
Healthy	6	F	23	6
Healthy	9	F	17	6
Healthy	8	F	11	4
Healthy	8	M	7	5
Healthy	5	F	5	6
Healthy	6	F	6	6
Healthy	4	M	13	7
Healthy	3	M	23	3
Healthy	3	M	30	6
Healthy	2	M	37	5
Diabetic	10	F	3	6
Diabetic	8	M	7	6
Diabetic	8	F	15	8
Diabetic	8	F	10	2
Diabetic	14	M	8	7
Diabetic	13	F	12	9
Diabetic	6	F	45	6
Diabetic	8	F	35	3
Diabetic	8	M	32	4
Diabetic	11	M	14	7
**Parameter**		**Healthy dogs**		**Diabetic dogs**
**B. CANINE GROUP COMPARISONS**				
No. of females/no. of males		5/5		6/4
Mean age (years)		5.4		9.4^*^
Mean weight (kg)		8.0		17.4
Mean BCS (0–9)		5.4		5.8

* *p < 0.05; BCS, body condition score*.

The different diets fed to HC and DM dogs were dictated by their overall health status and individual owner preference. For example, the majority (*n* = 7) of DM dogs were obese (i.e., body condition score [BCS] > 5) and therefore were placed on low-fat, high-fiber (i.e., standard) weight-reducing commercial rations. Those DM dogs having normal body condition (*n* = 3) were fed canine commercial-derived maintenance rations which do not promote weight loss when fed at recommended levels. The HC dogs having normal body condition (*n* = 5) were also fed commercial maintenance rations while 3 HC dogs having an obese phenotype were fed low-fat weight-reducing diets. Two HC dogs with past histories of adverse food reaction were currently fed commercial elimination diets at the time of enrollment.

**Figure 1:**
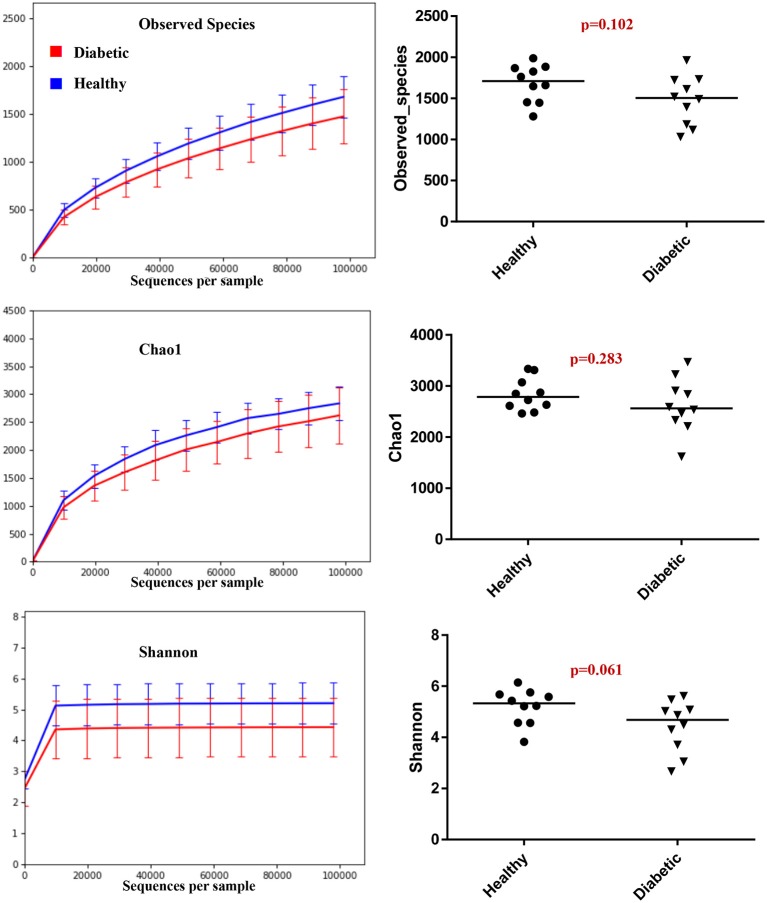
Summary of alpha diversity measures.

**Figure 2:**
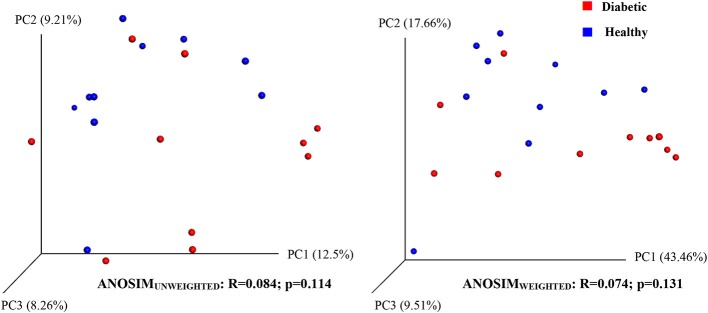
Principal coordinate analysis (PCoA) plot showing clustering of microbial communities from feces of healthy and diabetic dogs (red = diabetic, blue = healthy). The microbiome (beta diversity) of healthy dogs did not differ from that of diabetic dogs (ANOSIM; *p* = 0.114).

**Figure 3:**
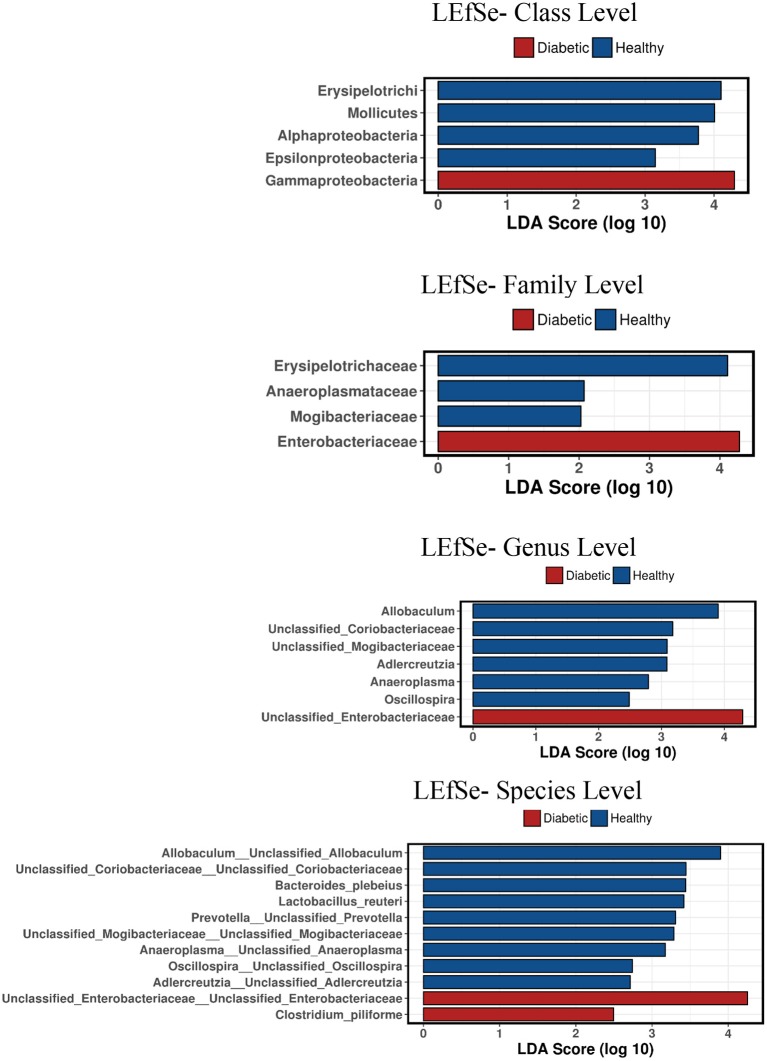
Linear discriminant analysis effect size (LefSe) of bacterial taxa and their association with different canine groups. Only LefSe values >2 are shown.

Analysis of bacterial communities showed no significant difference for any of the alpha-diversity measures between DM dogs vs. HC dogs (Figure 1). Principal coordinate analysis (PCA) based on unweighted Unifrac distance metric did not reveal significant clustering between dog groups (ANOSIM _Unweighted_: *R* = 0.084, *p* = 0.114; ANOSIM_weighted_: *R* = 0.074, *p* = 0.131; Figure 2). Calculation of Bray-Curtis dissimilarity was significant (i.e., *R* = 0.132, *p* = 0.038). PCA based on unweighted Unifrac distance metric was compared using ANOSIM to address confounding factors, such as administration of antibiotics, body condition score, diet, concurrent health issues, and sexual status. None of these comparisons reached statistical significance between comparison groups. However, linear discriminate analysis effects size (LEfSe) detected differentially abundant bacterial taxa (α = 0.01, LDA score >2.0) on various phylogenetic levels (Figure 3). While the family *Enterobacteriaceae* was overrepresented in dogs with DM, the proportions of the class Erysipelotrichia, and families *Mogibacteriaceae*, and *Anaeroplasmataceae* were increased in HC dogs. Similarly, on a species level, the abundance of an unclassified species belonging to *Enterobacteriaceae* family was most strongly associated with dogs having DM, while the abundance of *Lactobacillus reuteri* and *Bacteroides plebeius* were most strongly associated with HC dogs. Univariate analysis confirmed these findings, as the same bacterial groups were significantly (*p* < 0.05) altered (Supplemental Table 1).

**Figure 4:**
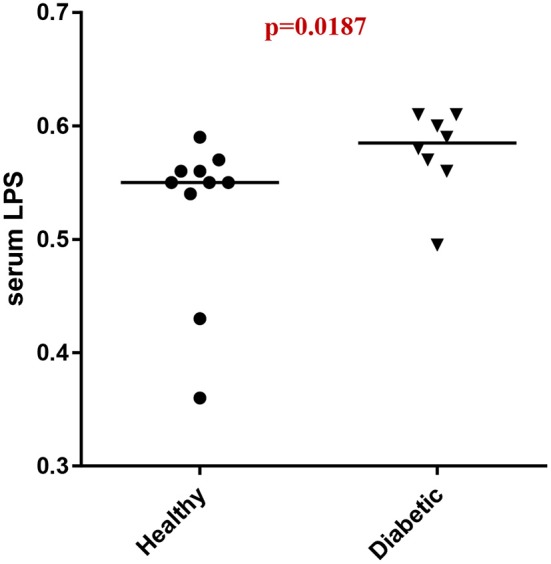
Serum LPS concentrations (EU/ml) in healthy controls (HC) vs. dogs with diabetes mellitus (DM).

Analysis of serum LPS concentrations showed that DM dogs had increased (*p* = 0.0187) circulatory levels of LPS vs. healthy dogs (Figure 4).

**Figure 5:**
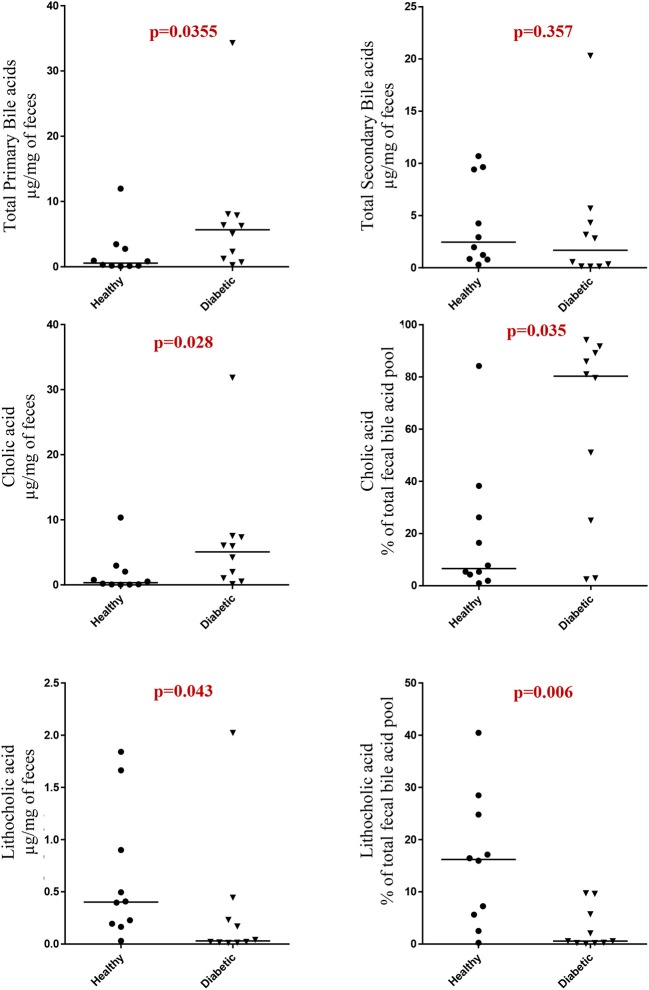
Comparison of primary and secondary fecal unconjugated bile acids in canine cohorts.

Dogs with DM had increased concentration of total primary fUBA compared to HC dogs (*p* = 0.028; Table 2). The concentrations of CA and the CA percentage of the total fUBA were increased (*p* = 0.029 and *p* = 0.036, respectively) in the feces of DM dogs relative to HC dogs. The levels of LCA (both absolute value and percentage of the total fUBA) were decreased (*p* = 0.043 and *p* = 0.01, respectively) in DM dogs vs. HC dogs (Figure 5).

**Table 2:** Fecal bile acid parameters between cohorts.

**Bile acid parameter**	***p*-Value**
Cholic acid	0.1053
Chenodeoxycholic acid	0.3027
Lithocholic acid	0.0072
Deoxycholic acid	0.0657
Ursodeoxycholic acid	0.3385
Total BA	0.6063
Total primary BA	0.0976
Total secondary BA	0.0772
Secondary to primary ratio	0.0429
Primary to secondary ratio	0.0429
Cholic acid % of Total	0.0510
Chenodeoxycholic acid % of Total	0.8829
Lithocholic acid % of Total	0.0036
Deoxycholic acid % of Total	0.2538
Ursodeoxycholic acid % of Total	0.1851
Total primary BA % of Total	0.0429
Total secondary BA% of Total	0.0429

*Shaded value indicates a fUBA of p < 0.05 indicating significant difference between canine groups. fUBA, fecal unconjugated bile acid*.

## Discussion

Alterations in gut microbiota composition have been linked to the development of human metabolic diseases, including both T1DM and T2DM (2, 3, 24, 25). The physiologic interplay between the intestinal microbiota and BA metabolism indicates that dysbiosis may be accompanied by altered BA homeostasis, thereby contributing to the metabolic dysregulation seen in DM. Dogs develop naturally-occurring, insulin-dependent DM which may also be associated with dysbiosis and altered bile acid metabolism, suggesting that they may serve as a clinically relevant model for investigating human disease.

We have now demonstrated that canine insulin-dependent DM is associated with changes in the composition of the intestinal microbiota evident from the family to species levels. In performing LEfSe analysis, we detected numerous bacterial taxa that were differentially abundant (e.g., LDA score >2.0) between dog groups. This included the relative abundance of family *Enterobacteriaceae* which was overrepresented while the abundance of subclass Erysipelotrichia, and of families *Mogibacteriaceae*, and *Anaeroplasmataceae* were underrepresented in DM dogs as compared to HC dogs. In support of our findings, other animal studies have shown that type 2 diabetic mice harbor reduced abundance of Bacteroides-related bacteria that are linked to endotoxemia-induced inflammation. Of interest, separate studies involving T2DM patients have shown differences in the relative abundance of select bacterial groups including the Bacteroidetes, Proteobacteria, and *Clostridia* spp (3, 26). While differences in gut microbial composition of human T2DM patients were observed between these different studies, one consistent finding was a decreased abundance of butyrate-producing bacteria (3, 27–29). This inconsistency regarding which bacteria are significantly altered in T2DM was later explained by the administration of metformin, a commonly administered glucose lowering drug (30).

It is now well-recognized that changes in the gut microbiota composition contribute to the development of metabolic endotoxemia (2, 24, 31). Specific compositional changes, caused by enrichment of Gram-negative species, may modulate host inflammatory activity through increased lipopolysaccharide (LPS) absorption. Importantly, the DM dogs in our study were observed to have increased abundance of Gammaproteobacteria and elevated serum LPS concentrations, similar to humans with T2DM (3). The LPS component of Gram negative bacteria acts as an immunodominant antigen which binds to toll-like receptor 4 (TLR-4) to trigger immune system activation (32–34) which has been previously reported in dogs (35). Interestingly, compared to HC dogs, serum LPS concentrations in DM dogs of the present study were elevated supporting the presence of metabolic endotoxemia. While we anticipated even higher levels of LPS to be present in DM dogs, the magnitude of serum elevation may have been influenced by the fact that most (7/10) DM dogs were being fed low-fat, weight reducing rations at the time of trial enrollment (36).

Bile acids have been previously investigated for their role in metabolic homeostasis and also for their well-known functions in lipid digestion (37). Distinct patterns of altered BA metabolism have been observed in rodent models and humans with T2DM, including increased fasting taurine-conjugated BA (T-BA) and post-prandial total BA responses (38, 39), increased urinary BA excretion (40), perturbations in serum BA metabolomic signature (41), and changes in bile acid metabolism present in non-obese, spontaneously diabetic (NOD) mice (42). In our study, we observed changes in both primary and secondary fUBA in insulin-dependent DM dogs, characterized by increased total primary fUBA with increased concentrations of CA and the CA percentage of the total fUBA, while the levels of LCA (both absolute value and percentage of the total fUBA) were decreased in DM dogs vs. HC dogs. Whereas, our data show similarities (43) and differences (13) to studies performed in humans, they all imply a distinct link between impaired glucose homeostasis and altered BA pool composition in diabetic-susceptible individuals.

The relationship between host-microbiota interactions and bile acids is complex and bidirectional. Microbial metabolism of BA involves a number of reactions including BA deconjugation by species having bile salt hydrolase (BSH) activity, 7-dehydroxylation of primary BA into secondary BA, and the generation of iso-BA (i.e., oxo- or keto-BA) by bacteria containing hydroxysteroid dehydrogenases (HSDHs) (44–50). As a result of altered BA composition, different BA modulate downstream signaling events through activation of receptors FXR and TGR5 in metabolically active tissues (51). In contrast, BA can modulate composition of the gut microbiota through their direct antimicrobial actions (i.e., destroying bacterial membranes by means of detergent properties) and indirectly through FXR activation which promotes transcription of antimicrobial (i.e., iNOS, IL-18) products (52). The ingestion of high fat diets may also impact host health by modulating BA and fecal microbiota composition to cause dysbiosis and local (gut mucosal) and systemic inflammation (53, 54).

Surprisingly, data investigating the role of gut microbiota in modulating the circulating BA pool in diabetic humans is sparse. A single study has analyzed the expression of microbiota-derived bile acid modification genes in humans with inflammatory bowel disease and diabetes (55). It was shown that Firmicute-derived bile salt hydrolase (BSH) genes and other BA modification genes were significantly reduced in the feces of T2DM patients relative to healthy controls. Similarly, other reports in animal models and humans with T2DM have shown the significance of gut microbial BSH genes in promoting positive physiologic changes and alteration in the overall BA pool (38, 56, 57). In one recent untargeted metabolomic study involving diabetic dogs, the primary and secondary bile acids, taurochenodeoxycholic acid, taurodeoxycholic acid, and tauroursodeoxycholic acid, were significantly lower in dogs with diabetes as compared to healthy dogs (58). While we observed reduced abundance of Firmicutes in the feces of our DM dogs, analysis of microbial-derived BSH or other BA modulating genes was not performed in this study.

There are only few published studies investigating canine BA profiles in health and disease. Most recent data suggest a relevant role for altered BA in canine chronic enteropathies. Honneffer et al. (59) utilized an untargeted metabolomic approach to identify several bile acid metabolites that were altered in the feces of dogs diagnosed with idiopathic inflammatory bowel disease (IBD). In a follow-up study to this report, Guard et al. (60) performed longitudinal assessment of microbial dysbiosis, fUBA concentrations and clinical disease activity in dogs with IBD that were treated with glucocorticoids. In that study, secondary fUBA were significantly decreased in IBD dogs and were accompanied by fecal dysbiosis and increased disease activity. While both fecal concentrations and the percentage of secondary fUBA increased post-treatment in IBD dogs, fecal microbial imbalances persisted in spite of resolution of clinical disease activity. Similarly, humans with IBD may have decreased proportions of fecal secondary BA without significantly altered primary BA (61) and increased primary BA are reported in people with diarrhea-predominant irritable bowel syndrome (62).

The ingestion of a high-fat diet (i.e., 75% of total energy requirement supplied by dietary fat) by healthy dogs has resulted in changes in fecal BA concentrations (63). In this pilot study, transitioning from a commercial dry food to a high-fat/low-fiber ration during a 7 weeks dietary trial increased fecal concentrations of the secondary BA DCA and UDCA. The investigators noted that there was significantly higher relative abundance of an OTU in the family *Clostridiaceae* (i.e., *Clostridia hiranonis*) which is particularly adept at converting primary BA to secondary BA. The consequences of this and other diet-associated alterations of the fecal microbiome and BA metabolome in dogs will require future adequately powered studies. There are some limitations to this study. First, the number of patients evaluated in both dog groups was relatively small and a longitudinal study design evaluating changes in microbiota and BA in response to treatment should be performed in the future. Trial enrollment of dogs with DM proved challenging due to the frequent use of antimicrobials in these patients (for suspicion of bacterial urinary tract infection or other unrelated causes) that reduced our sample size. Second, both cohorts were not fed a standardized diet which may have influenced microbial composition and the production of secondary BA. Third, we report data only for fUBA (but not conjugated BA) which may not provide a complete picture of BA dysmetabolism present in dogs with DM. That said, we are unaware of other published methods, beyond those described in the present study, to measure fecal BA concentrations in healthy and diseased dogs.

In conclusion, our results indicate that both intestinal dysbiosis and altered fecal BA levels are present in dogs with naturally occurring, insulin-dependent DM. Diabetic dogs demonstrate increased fecal primary (CA) BA and decreased levels of secondary (LCA) BA. The patterns of microbial imbalance and impaired BA homeostasis bear strong similarity to T2DM in humans. The dog represents a novel large animal model for advancing translational medicine research efforts (e.g., investigating pathogenesis and therapeutics) in DM affecting humans.

## Author Contributions

AJ, BG, AR, and JoS contributed to research design and implementation. AJ, BG, AR, JaS, GR, and LC performed the experiments. AJ, BG JaS, JM, LC, YS, and RP contributed to data analysis. AJ, BG, JM, YS, JaS, and RP prepared the manuscript. AJ, BG, JaS, JM, JoS, KA, JL, and RP reviewed and edited the manuscript.

### Conflict of Interest Statement

The authors declare that the research was conducted in the absence of any commercial or financial relationships that could be construed as a potential conflict of interest.

## Footnotes

^1^Hill's prescription diet w/d.

^2^Purina Pro Plan Veterinary Diet OM.

## Supplementary Material

Supplemental Table 1 Percentage of bacterial taxa.
