# Supplementary material for: Microbiota-Related Changes in Unconjugated Fecal Bile Acids Are Associated With Naturally Occurring, Insulin-Dependent Diabetes Mellitus in Dogs
Source: Front Vet Sci. 2019 Jun 27;6:199. doi: 10.3389/fvets.2019.00199 (PMC6610424; doi:10.3389/fvets.2019.00199)
Supplement: Supplemental Table 1 — Percentage of bacterial taxa. [file Table_1.docx]

| Supplemental Table 1 - Bacterial taxa |  |  |  |  |  |  |  |  |  |
| --- | --- | --- | --- | --- | --- | --- | --- | --- | --- |
| **Phylum** |  | **Healthy** | |  | **Diabetic** | |  | **Healthy vs Diabetic** | |
|  |  |  |  |  |  |  |  |  |  |
|  |  |  |  |  |  |  |  |  |  |
|  |  | **Median** | **Range** |  | **Median** | **Range** |  | **P value** | **Q value** |
|  |  |  |  |  |  |  |  |  |  |
| Teneicutes |  | 0 | 0-0 |  | 0 | 0-0 |  | 0.046 | 0.228 |
| Protobacteria |  | 0.4 | 0.2-10.9 |  | 1.5 | 0.3-12.2 |  | 0.096 | 0.321 |
| Actiobacteria |  | 4.1 | 0.2-18.2 |  | 2.3 | 0.1-8.7 |  | 0.151 | 0.377 |
| Euryachaeota |  | 0 | 0-0.1 |  | 0 | 0-0 |  | 0.583 | 0.746 |
| Bactroidetes |  | 3.9 | 0.2-26.7 |  | 1.9 | 0.2-23.3 |  | 0.597 | 0.746 |
| Fusoacteria |  | 3.6 | 0.2-21.5 |  | 1.7 | 0.1-37.1 |  | 0.545 | 0.746 |
| Verrcomicrobia |  | 0 | 0-0 |  | 0 | 0-0 |  | 0.84 | 0.933 |
| Firmcutes |  | 86.6 | 33.8-96.6 |  | 83.8 | 47-98.5 |  | 0.94 | 0.94 |
|  |  |  |  |  |  |  |  |  |  |
|  |  |  |  |  |  |  |  |  |  |
| **Class** |  | **Healthy** | |  | **Diabetic** | |  | **Healthy vs Diabetic** | |
|  |  |  |  |  |  |  |  |  |  |
|  |  |  |  |  |  |  |  |  |  |
|  |  | **Median** | **Range** |  | **Median** | **Range** |  | **P value** | **Q value** |
|  |  |  |  |  |  |  |  |  |  |
| Gammaproteobacteria |  | 0.1 | 0-0.4 |  | 1 | 0-12.1 |  | 0.005 | 0.088 |
| Erysipelotrichi |  | 1.9 | 0.2-13.9 |  | 0.5 | 0-4.5 |  | 0.034 | 0.122 |
| Alphaproteobacteria |  | 0 | 0-0.1 |  | 0 | 0-0 |  | 0.019 | 0.122 |
| Epsilonproteobacteria |  | 0.1 | 0-0.8 |  | 0 | 0-1 |  | 0.028 | 0.122 |
| Mollicutes |  | 0 | 0-0 |  | 0 | 0-0 |  | 0.046 | 0.129 |
| Actinobacteria |  | 0.2 | 0.1-16.2 |  | 0.2 | 0-3.1 |  | 0.241 | 0.515 |
| Coriobacteriia |  | 2.5 | 0.1-8.5 |  | 1 | 0-8.6 |  | 0.257 | 0.515 |
| Betaproteobacteria |  | 0.1 | 0-10.6 |  | 0.1 | 0-0.5 |  | 0.273 | 0.515 |
| Bacilli |  | 21.2 | 0.4-60 |  | 26.9 | 0.6-65.4 |  | 0.364 | 0.516 |
| Deltaproteobacteria |  | 0 | 0-0.1 |  | 0 | 0-0 |  | 0.355 | 0.516 |
| Methanobacteria |  | 0 | 0-0.1 |  | 0 | 0-0 |  | 0.583 | 0.676 |
| Bacteroidia |  | 3.9 | 0.2-26.7 |  | 1.9 | 0.2-23.3 |  | 0.597 | 0.676 |
| Fusobacteriia |  | 3.6 | 0.2-21.5 |  | 1.7 | 0.1-37.1 |  | 0.545 | 0.676 |
| Clostridia |  | 56.1 | 15.5-89.5 |  | 50.1 | 23.5-72.5 |  | 0.762 | 0.81 |
| Verrucomicrobiae |  | 0 | 0-0 |  | 0 | 0-0 |  | 0.936 | 0.936 |
|  |  |  |  |  |  |  |  |  |  |
|  |  |  |  |  |  |  |  |  |  |
| **Order** |  | **Healthy** | |  | **Diabetic** | |  | **Healthy vs Diabetic** | |
|  |  |  |  |  |  |  |  |  |  |
|  |  |  |  |  |  |  |  |  |  |
|  |  | **Median** | **Range** |  | **Median** | **Range** |  | **P value** | **Q value** |
|  |  |  |  |  |  |  |  |  |  |
| Erysipelotrichales |  | 1.9 | 0.2-13.9 |  | 0.5 | 0-4.5 |  | 0.034 | 0.198 |
| Campylobacterales |  | 0.1 | 0-0.8 |  | 0 | 0-1 |  | 0.028 | 0.198 |
| Enterobacteriales |  | 0.1 | 0-0.4 |  | 0.7 | 0-12.1 |  | 0.023 | 0.198 |
| Anaeroplasmatales |  | 0 | 0-0 |  | 0 | 0-0 |  | 0.046 | 0.2 |
| Rickettsiales |  | 0 | 0-0.1 |  | 0 | 0-0 |  | 0.078 | 0.287 |
| Bifidobacteriales |  | 0.2 | 0.1-16.1 |  | 0.1 | 0-3 |  | 0.29 | 0.668 |
| Coriobacteriales |  | 2.5 | 0.1-8.5 |  | 1 | 0-8.6 |  | 0.257 | 0.668 |
| Lactobacillales |  | 7.1 | 0.4-59.9 |  | 26.8 | 0.3-65.3 |  | 0.364 | 0.668 |
| Burkholderiales |  | 0.1 | 0-10.4 |  | 0.1 | 0-0.5 |  | 0.29 | 0.668 |
| Desulfovibrionales |  | 0 | 0-0.1 |  | 0 | 0-0 |  | 0.355 | 0.668 |
| Methanobacteriales |  | 0 | 0-0.1 |  | 0 | 0-0 |  | 0.583 | 0.772 |
| Bacteroidales |  | 3.9 | 0.2-26.7 |  | 1.9 | 0.2-23.3 |  | 0.597 | 0.772 |
| Bacillales |  | 0 | 0-0.1 |  | 0 | 0-0 |  | 0.595 | 0.772 |
| Fusobacteriales |  | 3.6 | 0.2-21.5 |  | 1.7 | 0.1-37.1 |  | 0.545 | 0.772 |
| Aeromonadales |  | 0 | 0-0.1 |  | 0 | 0-1.7 |  | 0.57 | 0.772 |
| Actinomycetales |  | 0 | 0-0.1 |  | 0 | 0-0.1 |  | 0.705 | 0.839 |
| Gemellales |  | 0 | 0-0 |  | 0 | 0-0 |  | 0.745 | 0.839 |
| Clostridiales |  | 56.1 | 15.5-89.5 |  | 50.1 | 23.5-72.5 |  | 0.762 | 0.839 |
| Turicibacterales |  | 0.1 | 0-15.8 |  | 0.2 | 0.1-2 |  | 0.97 | 0.97 |
| Verrucomicrobiales |  | 0 | 0-0 |  | 0 | 0-0 |  | 0.936 | 0.97 |
|  |  |  |  |  |  |  |  |  |  |
|  |  |  |  |  |  |  |  |  |  |
| **Family** |  | **Healthy** | |  | **Diabetic** | |  | **Healthy vs Diabetic** | |
|  |  |  |  |  |  |  |  |  |  |
|  |  |  |  |  |  |  |  |  |  |
|  |  | **Median** | **Range** |  | **Median** | **Range** |  | **P value** | **Q value** |
|  |  |  |  |  |  |  |  |  |  |
| Mogibacteriaceae |  | 0 | 0-0.1 |  | 0 | 0-0 |  | 0.052 | 0.397 |
| Erysipelotrichaceae |  | 1.9 | 0.2-13.9 |  | 0.5 | 0-4.5 |  | 0.034 | 0.397 |
| Campylobacteraceae |  | 0 | 0-0.1 |  | 0 | 0-0 |  | 0.019 | 0.397 |
| Enterobacteriaceae |  | 0.1 | 0-0.4 |  | 0.7 | 0-12.1 |  | 0.023 | 0.397 |
| Anaeroplasmataceae |  | 0 | 0-0 |  | 0 | 0-0 |  | 0.046 | 0.397 |
| Veillonellaceae |  | 0.5 | 0.1-1.9 |  | 0.1 | 0-14.5 |  | 0.07 | 0.4 |
| Helicobacteraceae |  | 0.1 | 0-0.8 |  | 0 | 0-1 |  | 0.069 | 0.4 |
| Paraprevotellaceae |  | 0 | 0-3.5 |  | 0 | 0-0 |  | 0.111 | 0.465 |
| o__Clostridiales.f__ |  | 0.4 | 0.1-0.7 |  | 0.2 | 0-2.3 |  | 0.104 | 0.465 |
| Eubacteriaceae |  | 0 | 0-0.2 |  | 0 | 0-0 |  | 0.108 | 0.465 |
| Bacillaceae |  | 0 | 0-0 |  | 0 | 0-0 |  | 0.135 | 0.509 |
| Lactobacillaceae |  | 0.2 | 0.1-44.8 |  | 0.2 | 0.1-1.6 |  | 0.151 | 0.509 |
| mitochondria |  | 0 | 0-0.1 |  | 0 | 0-0 |  | 0.155 | 0.509 |
| Staphylococcaceae |  | 0 | 0-0 |  | 0 | 0-0 |  | 0.188 | 0.54 |
| Streptococcaceae |  | 2.4 | 0.1-59.3 |  | 26.3 | 0.1-65.1 |  | 0.186 | 0.54 |
| o__Clostridiales.Other |  | 0 | 0-0.2 |  | 0 | 0-0.1 |  | 0.212 | 0.542 |
| Desulfovibrionaceae |  | 0 | 0-0.1 |  | 0 | 0-0 |  | 0.211 | 0.542 |
| Bifidobacteriaceae |  | 0.2 | 0.1-16.1 |  | 0.1 | 0-3 |  | 0.29 | 0.565 |
| Coriobacteriaceae |  | 2.5 | 0.1-8.5 |  | 1 | 0-8.6 |  | 0.257 | 0.565 |
| Aerococcaceae |  | 0 | 0-0 |  | 0 | 0-0 |  | 0.307 | 0.565 |
| Peptococcaceae |  | 0 | 0-0.3 |  | 0 | 0-0.3 |  | 0.281 | 0.565 |
| Tissierellaceae |  | 0 | 0-0 |  | 0 | 0-0 |  | 0.244 | 0.565 |
| Alcaligenaceae |  | 0.1 | 0-10.4 |  | 0.1 | 0-0.5 |  | 0.29 | 0.565 |
| Peptostreptococcaceae |  | 0.8 | 0.1-7.8 |  | 1.2 | 0.1-11.9 |  | 0.326 | 0.576 |
| Prevotellaceae |  | 0.1 | 0-0.3 |  | 0 | 0-7.5 |  | 0.345 | 0.578 |
| S24.7 |  | 0 | 0-11.3 |  | 0 | 0-0 |  | 0.362 | 0.578 |
| Clostridiaceae |  | 18.6 | 0.4-45.5 |  | 5 | 0.4-57 |  | 0.364 | 0.578 |
| Ruminococcaceae |  | 0.2 | 0.1-3.7 |  | 0.2 | 0-0.9 |  | 0.427 | 0.655 |
| Methanobacteriaceae |  | 0 | 0-0.1 |  | 0 | 0-0 |  | 0.583 | 0.789 |
| Rikenellaceae |  | 0 | 0-0 |  | 0 | 0-0 |  | 0.559 | 0.789 |
| Fusobacteriaceae |  | 3.6 | 0.2-21.5 |  | 1.7 | 0.1-37.1 |  | 0.545 | 0.789 |
| Succinivibrionaceae |  | 0 | 0-0.1 |  | 0 | 0-1.7 |  | 0.57 | 0.789 |
| Actinomycetaceae |  | 0 | 0-0 |  | 0 | 0-0.1 |  | 0.762 | 0.857 |
| Bacteroidaceae |  | 3.2 | 0.1-17.6 |  | 0.5 | 0.2-23.1 |  | 0.706 | 0.857 |
| Gemellaceae |  | 0 | 0-0 |  | 0 | 0-0 |  | 0.745 | 0.857 |
| o__Lactobacillales.Other |  | 0 | 0-0 |  | 0 | 0-0 |  | 0.732 | 0.857 |
| Enterococcaceae |  | 0.1 | 0.1-4.3 |  | 0.1 | 0.1-1.5 |  | 0.762 | 0.857 |
| Leuconostocaceae |  | 0 | 0-0.1 |  | 0 | 0-0 |  | 0.764 | 0.857 |
| Lachnospiraceae |  | 23.4 | 7.7-83.6 |  | 29.6 | 7.5-52.8 |  | 0.762 | 0.857 |
| Microbacteriaceae |  | 0 | 0-0 |  | 0 | 0-0 |  | 0.848 | 0.929 |
| Corynebacteriaceae |  | 0 | 0-0.1 |  | 0 | 0-0 |  | 0.97 | 0.97 |
| Porphyromonadaceae |  | 0 | 0-1.2 |  | 0 | 0-0.2 |  | 0.97 | 0.97 |
| Turicibacteraceae |  | 0.1 | 0-15.8 |  | 0.2 | 0.1-2 |  | 0.97 | 0.97 |
| Verrucomicrobiaceae |  | 0 | 0-0 |  | 0 | 0-0 |  | 0.936 | 0.97 |
|  |  |  |  |  |  |  |  |  |  |
|  |  |  |  |  |  |  |  |  |  |
| **Genus** |  | **Healthy** | |  | **Diabetic** | |  | **Healthy vs Diabetic** | |
|  |  |  |  |  |  |  |  |  |  |
|  |  |  |  |  |  |  |  |  |  |
|  |  | **Median** | **Range** |  | **Median** | **Range** |  | **P value** | **Q value** |
|  |  |  |  |  |  |  |  |  |  |
| Clostridium |  | 0 | 0-0 |  | 0 | 0-0.2 |  | 0.016 | 0.377 |
| f__.Mogibacteriaceae..g__ |  | 0 | 0-0.1 |  | 0 | 0-0 |  | 0.018 | 0.377 |
| Campylobacter |  | 0 | 0-0.1 |  | 0 | 0-0 |  | 0.019 | 0.377 |
| f__Enterobacteriaceae.Other |  | 0 | 0-0 |  | 0 | 0-0 |  | 0.012 | 0.377 |
| f__Enterobacteriaceae.g__ |  | 0.1 | 0-0.4 |  | 0.7 | 0-12.1 |  | 0.023 | 0.377 |
| Adlercreutzia |  | 0 | 0-0.2 |  | 0 | 0-0 |  | 0.04 | 0.436 |
| Oscillospira |  | 0 | 0-0.2 |  | 0 | 0-0.1 |  | 0.048 | 0.436 |
| Anaeroplasma |  | 0 | 0-0 |  | 0 | 0-0 |  | 0.046 | 0.436 |
| f__Clostridiaceae.Other |  | 9.6 | 0.1-30.2 |  | 1.5 | 0.1-22.6 |  | 0.059 | 0.449 |
| Proteus |  | 0 | 0-0 |  | 0 | 0-0 |  | 0.061 | 0.449 |
| Prevotella |  | 0 | 0-2.1 |  | 0 | 0-0 |  | 0.088 | 0.52 |
| Megamonas |  | 0.3 | 0-1.9 |  | 0.1 | 0-14.5 |  | 0.096 | 0.52 |
| Allobaculum |  | 0.1 | 0-13.9 |  | 0 | 0-0.2 |  | 0.095 | 0.52 |
| Helicobacter |  | 0.1 | 0-0.8 |  | 0 | 0-1 |  | 0.082 | 0.52 |
| o__Clostridiales.f__.g__ |  | 0.4 | 0.1-0.7 |  | 0.2 | 0-2.3 |  | 0.104 | 0.527 |
| f__Coriobacteriaceae.g__ |  | 0 | 0-2.3 |  | 0 | 0-0 |  | 0.113 | 0.536 |
| Slackia |  | 0 | 0-0.2 |  | 0 | 0-0.2 |  | 0.134 | 0.571 |
| Lactobacillus |  | 0.2 | 0.1-44.8 |  | 0.2 | 0.1-1.6 |  | 0.131 | 0.571 |
| Faecalibacterium |  | 0.1 | 0-3 |  | 0 | 0-0.6 |  | 0.151 | 0.571 |
| f__Erysipelotrichaceae.g__ |  | 0.4 | 0-2.2 |  | 0.1 | 0-4.4 |  | 0.151 | 0.571 |
| f__mitochondria.Other |  | 0 | 0-0.1 |  | 0 | 0-0 |  | 0.155 | 0.571 |
| f__Lachnospiraceae.Other |  | 0.3 | 0.2-3.7 |  | 0.3 | 0.1-0.7 |  | 0.174 | 0.611 |
| Streptococcus |  | 2.4 | 0.1-59.3 |  | 26.3 | 0.1-65.1 |  | 0.186 | 0.627 |
| Staphylococcus |  | 0 | 0-0 |  | 0 | 0-0 |  | 0.215 | 0.633 |
| o__Clostridiales.Other.Other |  | 0 | 0-0.2 |  | 0 | 0-0.1 |  | 0.212 | 0.633 |
| f__Clostridiaceae.g__ |  | 7.2 | 0.2-15.1 |  | 1.8 | 0.2-16.7 |  | 0.227 | 0.633 |
| Clostridium |  | 0.3 | 0-13.6 |  | 0.1 | 0-54.5 |  | 0.227 | 0.633 |
| f__Ruminococcaceae.g__ |  | 0.1 | 0-0.9 |  | 0 | 0-0.3 |  | 0.227 | 0.633 |
| Catenibacterium |  | 0 | 0-5.2 |  | 0 | 0-0.2 |  | 0.24 | 0.649 |
| Bifidobacterium |  | 0.2 | 0.1-16.1 |  | 0.1 | 0-3 |  | 0.29 | 0.652 |
| Peptococcus |  | 0 | 0-0.3 |  | 0 | 0-0.3 |  | 0.281 | 0.652 |
| Dialister |  | 0 | 0-0 |  | 0 | 0-0 |  | 0.289 | 0.652 |
| Coprobacillus |  | 0 | 0-0.2 |  | 0 | 0-0 |  | 0.29 | 0.652 |
| Eubacterium |  | 0.1 | 0-2.5 |  | 0.1 | 0-2.5 |  | 0.29 | 0.652 |
| Sutterella |  | 0.1 | 0-10.4 |  | 0.1 | 0-0.5 |  | 0.29 | 0.652 |
| f__Aerococcaceae.g__ |  | 0 | 0-0 |  | 0 | 0-0 |  | 0.307 | 0.655 |
| Pediococcus |  | 0 | 0-0 |  | 0 | 0-0 |  | 0.316 | 0.655 |
| f__Lachnospiraceae.g__ |  | 2 | 1.2-5.8 |  | 1.4 | 0.2-7.4 |  | 0.326 | 0.66 |
| Prevotella |  | 0.1 | 0-0.3 |  | 0 | 0-7.5 |  | 0.345 | 0.681 |
| f__S24.7.g__ |  | 0 | 0-11.3 |  | 0 | 0-0 |  | 0.362 | 0.697 |
| Leucobacter |  | 0 | 0-0 |  | 0 | 0-0 |  | 0.39 | 0.714 |
| Coprococcus |  | 0.1 | 0.1-1.5 |  | 0.3 | 0.1-4.4 |  | 0.406 | 0.714 |
| f__Peptostreptococcaceae.g__ |  | 0.2 | 0-3 |  | 0.2 | 0.1-7.7 |  | 0.406 | 0.714 |
| Clostridium |  | 0 | 0-0 |  | 0 | 0-0 |  | 0.389 | 0.714 |
| Sarcina |  | 0 | 0-0 |  | 0 | 0-0 |  | 0.436 | 0.736 |
| f__Peptostreptococcaceae.Other |  | 0.2 | 0-4.9 |  | 0.5 | 0-8.2 |  | 0.427 | 0.736 |
| f__Fusobacteriaceae.Other |  | 0.1 | 0-0.3 |  | 0 | 0-0.6 |  | 0.45 | 0.743 |
| Collinsella |  | 2.1 | 0-8.2 |  | 1 | 0-8.4 |  | 0.496 | 0.758 |
| Lactobacillaceae.g__ |  | 0 | 0-0 |  | 0 | 0-0 |  | 0.471 | 0.758 |
| SMB53 |  | 0.1 | 0-0.2 |  | 0.1 | 0-0.1 |  | 0.496 | 0.758 |
| Dorea |  | 1.7 | 0.7-15.7 |  | 3.7 | 0.5-11.1 |  | 0.496 | 0.758 |
| Megasphaera |  | 0 | 0-0.6 |  | 0 | 0-0 |  | 0.545 | 0.817 |
| f__Rikenellaceae.g__ |  | 0 | 0-0 |  | 0 | 0-0 |  | 0.559 | 0.823 |
| Methanobrevibacter |  | 0 | 0-0.1 |  | 0 | 0-0 |  | 0.583 | 0.843 |
| f__Succinivibrionaceae.g__ |  | 0 | 0-0 |  | 0 | 0-1.6 |  | 0.611 | 0.868 |
| Fusobacterium |  | 3.4 | 0.2-9.6 |  | 1.7 | 0.1-36.6 |  | 0.65 | 0.908 |
| Actinomyces |  | 0 | 0-0 |  | 0 | 0-0.1 |  | 0.762 | 0.936 |
| Bacteroides |  | 3.2 | 0.1-17.6 |  | 0.5 | 0.2-23.1 |  | 0.706 | 0.936 |
| Parabacteroides |  | 0 | 0-1.2 |  | 0 | 0-0.2 |  | 0.704 | 0.936 |
| Gemella |  | 0 | 0-0 |  | 0 | 0-0 |  | 0.745 | 0.936 |
| o__Lactobacillales.Other.Other |  | 0 | 0-0 |  | 0 | 0-0 |  | 0.732 | 0.936 |
| Enterococcus |  | 0.1 | 0.1-4.3 |  | 0.1 | 0.1-1.5 |  | 0.762 | 0.936 |
| Blautia |  | 14.9 | 1.9-48 |  | 16.6 | 2.4-34.3 |  | 0.762 | 0.936 |
| Roseburia |  | 0 | 0-0 |  | 0 | 0-0 |  | 0.759 | 0.936 |
| Candidatus.Arthromitus |  | 0 | 0-0.1 |  | 0 | 0-2.1 |  | 0.778 | 0.941 |
| Ruminococcus |  | 0 | 0-0.1 |  | 0 | 0-0.1 |  | 0.791 | 0.942 |
| f__Enterococcaceae.Other |  | 0 | 0-0 |  | 0 | 0-0 |  | 0.843 | 0.948 |
| Lactococcus |  | 0 | 0-0 |  | 0 | 0-0.2 |  | 0.839 | 0.948 |
| Ruminococcus |  | 3.7 | 0.2-11.6 |  | 3.7 | 0.3-7.2 |  | 0.821 | 0.948 |
| Peptostreptococcus |  | 0 | 0-0 |  | 0 | 0-0 |  | 0.816 | 0.948 |
| Phascolarctobacterium |  | 0 | 0-0.3 |  | 0 | 0-0 |  | 0.878 | 0.961 |
| Cetobacterium |  | 0 | 0-12 |  | 0 | 0-0 |  | 0.876 | 0.961 |
| Corynebacterium |  | 0 | 0-0.1 |  | 0 | 0-0 |  | 0.97 | 0.97 |
| Turicibacter |  | 0.1 | 0-15.8 |  | 0.2 | 0.1-2 |  | 0.97 | 0.97 |
| Butyrivibrio |  | 0 | 0-0 |  | 0 | 0-0 |  | 0.967 | 0.97 |
| Epulopiscium |  | 0 | 0-1.5 |  | 0 | 0-0.1 |  | 0.97 | 0.97 |
| f__Ruminococcaceae.Other |  | 0 | 0-0 |  | 0 | 0-0 |  | 0.935 | 0.97 |
| Anaerobiospirillum |  | 0 | 0-0.1 |  | 0 | 0-0 |  | 0.908 | 0.97 |
| Akkermansia |  | 0 | 0-0 |  | 0 | 0-0 |  | 0.936 | 0.97 |
|  |  |  |  |  |  |  |  |  |  |
| **Species** |  | **Healthy** | |  | **Diabetic** | |  | **Healthy vs Diabetic** | |
|  |  |  |  |  |  |  |  |  |  |
|  |  |  |  |  |  |  |  |  |  |
|  |  | **Median** | **Range** |  | **Median** | **Range** |  | **P value** | **Q value** |
|  |  |  |  |  |  |  |  |  |  |
| Lactobacillus reuteri |  | 0 | 0-3.4 |  | 0 | 0-0 |  | 0.026 | 0.358 |
| Clostridium piliforme |  | 0 | 0-0 |  | 0 | 0-0.2 |  | 0.016 | 0.358 |
| g__[Ruminococcus];Other |  | 0 | 0-0 |  | 0 | 0-0 |  | 0.026 | 0.358 |
| [Ruminococcus] torques |  | 0 | 0-0 |  | 0 | 0-0 |  | 0.012 | 0.358 |
| f__[Mogibacteriaceae];g__;s__ |  | 0 | 0-0.1 |  | 0 | 0-0 |  | 0.018 | 0.358 |
| g__Campylobacter;s__ |  | 0 | 0-0.1 |  | 0 | 0-0 |  | 0.019 | 0.358 |
| f__Enterobacteriaceae;Other;Other |  | 0 | 0-0 |  | 0 | 0-0 |  | 0.012 | 0.358 |
| f__Enterobacteriaceae;g__;s__ |  | 0.1 | 0-0.4 |  | 0.7 | 0-12.1 |  | 0.023 | 0.358 |
| g__Adlercreutzia;s__ |  | 0 | 0-0.2 |  | 0 | 0-0 |  | 0.04 | 0.406 |
| Streptococcus anginosus |  | 0 | 0-0 |  | 0 | 0-0 |  | 0.045 | 0.406 |
| g__Oscillospira;s__ |  | 0 | 0-0.2 |  | 0 | 0-0.1 |  | 0.048 | 0.406 |
| g__Anaeroplasma;s__ |  | 0 | 0-0 |  | 0 | 0-0 |  | 0.046 | 0.406 |
| f__Clostridiaceae;Other;Other |  | 9.6 | 0.1-30.2 |  | 1.5 | 0.1-22.6 |  | 0.059 | 0.416 |
| Clostridium hiranonis |  | 0.1 | 0-0.1 |  | 0 | 0-0.1 |  | 0.058 | 0.416 |
| g__Proteus;s__ |  | 0 | 0-0 |  | 0 | 0-0 |  | 0.061 | 0.416 |
| Bacteroides plebeius |  | 0.2 | 0-2.3 |  | 0 | 0-0.4 |  | 0.069 | 0.441 |
| Bacteroides coprophilus |  | 0 | 0-0.5 |  | 0 | 0-0 |  | 0.09 | 0.472 |
| g__[Prevotella];s__ |  | 0 | 0-2.1 |  | 0 | 0-0 |  | 0.088 | 0.472 |
| o__Clostridiales;f__;g__;s__ |  | 0.4 | 0.1-0.7 |  | 0.2 | 0-2.3 |  | 0.104 | 0.472 |
| g__[Ruminococcus];s__ |  | 0.2 | 0-1.1 |  | 0 | 0-0.4 |  | 0.104 | 0.472 |
| g__Megamonas;s__ |  | 0.3 | 0-1.9 |  | 0.1 | 0-14.5 |  | 0.096 | 0.472 |
| g__Allobaculum;s__ |  | 0.1 | 0-13.9 |  | 0 | 0-0.2 |  | 0.095 | 0.472 |
| g__Helicobacter;s__ |  | 0.1 | 0-0.8 |  | 0 | 0-1 |  | 0.082 | 0.472 |
| f__Coriobacteriaceae;g__;s__ |  | 0 | 0-2.3 |  | 0 | 0-0 |  | 0.113 | 0.491 |
| g__Slackia;s__ |  | 0 | 0-0.2 |  | 0 | 0-0.2 |  | 0.134 | 0.562 |
| g__Lactobacillus;s__ |  | 0.2 | 0.1-41.4 |  | 0.1 | 0.1-1.5 |  | 0.151 | 0.563 |
| Faecalibacterium prausnitzii |  | 0.1 | 0-3 |  | 0 | 0-0.6 |  | 0.151 | 0.563 |
| f__Erysipelotrichaceae;g__;s__ |  | 0.4 | 0-2.2 |  | 0.1 | 0-4.4 |  | 0.151 | 0.563 |
| f__mitochondria;Other;Other |  | 0 | 0-0.1 |  | 0 | 0-0 |  | 0.155 | 0.563 |
| g__Streptococcus;s__ |  | 2.4 | 0.1-59.2 |  | 26.2 | 0.1-65 |  | 0.174 | 0.591 |
| f__Lachnospiraceae;Other;Other |  | 0.3 | 0.2-3.7 |  | 0.3 | 0.1-0.7 |  | 0.174 | 0.591 |
| g__Staphylococcus;s__ |  | 0 | 0-0 |  | 0 | 0-0 |  | 0.187 | 0.617 |
| g__Bifidobacterium;s__ |  | 0.1 | 0-1.2 |  | 0 | 0-0.2 |  | 0.198 | 0.636 |
| g__Lactobacillus;Other |  | 0 | 0-0 |  | 0 | 0-0 |  | 0.211 | 0.639 |
| Streptococcus minor |  | 0 | 0-0.1 |  | 0 | 0-0.1 |  | 0.239 | 0.639 |
| o__Clostridiales;Other;Other;Other |  | 0 | 0-0.2 |  | 0 | 0-0.1 |  | 0.212 | 0.639 |
| f__Clostridiaceae;g__;s__ |  | 7.2 | 0.2-15.1 |  | 1.8 | 0.2-16.7 |  | 0.227 | 0.639 |
| f__Ruminococcaceae;g__;s__ |  | 0.1 | 0-0.9 |  | 0 | 0-0.3 |  | 0.227 | 0.639 |
| g__Catenibacterium;s__ |  | 0 | 0-5.2 |  | 0 | 0-0.2 |  | 0.24 | 0.639 |
| [Eubacterium] biforme |  | 0 | 0-2.5 |  | 0 | 0-2.5 |  | 0.24 | 0.639 |
| Streptococcus luteciae |  | 0 | 0-0.1 |  | 0 | 0-0.1 |  | 0.289 | 0.658 |
| Clostridium perfringens |  | 0 | 0-13.3 |  | 0 | 0-0.2 |  | 0.273 | 0.658 |
| g__Blautia;Other |  | 1 | 0.1-2.2 |  | 0.4 | 0-2.8 |  | 0.29 | 0.658 |
| g__Peptococcus;s__ |  | 0 | 0-0.3 |  | 0 | 0-0.3 |  | 0.281 | 0.658 |
| g__Dialister;s__ |  | 0 | 0-0 |  | 0 | 0-0 |  | 0.289 | 0.658 |
| g__Coprobacillus;s__ |  | 0 | 0-0.2 |  | 0 | 0-0 |  | 0.29 | 0.658 |
| g__Sutterella;s__ |  | 0.1 | 0-10.4 |  | 0.1 | 0-0.5 |  | 0.29 | 0.658 |
| Bacteroides uniformis |  | 0 | 0-0.1 |  | 0 | 0-0.1 |  | 0.319 | 0.681 |
| f__Aerococcaceae;g__;s__ |  | 0 | 0-0 |  | 0 | 0-0 |  | 0.307 | 0.681 |
| g__Pediococcus;s__ |  | 0 | 0-0 |  | 0 | 0-0 |  | 0.316 | 0.681 |
| f__Lachnospiraceae;g__;s__ |  | 2 | 1.2-5.8 |  | 1.4 | 0.2-7.4 |  | 0.326 | 0.683 |
| Prevotella copri |  | 0.1 | 0-0.3 |  | 0 | 0-7.5 |  | 0.345 | 0.709 |
| f__S24-7;g__;s__ |  | 0 | 0-11.3 |  | 0 | 0-0 |  | 0.362 | 0.721 |
| g__Streptococcus;Other |  | 0 | 0-0.1 |  | 0 | 0-0.1 |  | 0.364 | 0.721 |
| g__Leucobacter;s__ |  | 0 | 0-0 |  | 0 | 0-0 |  | 0.39 | 0.737 |
| g__Bacteroides;s__ |  | 2.4 | 0.1-17.5 |  | 0.3 | 0.1-14 |  | 0.406 | 0.737 |
| g__Coprococcus;s__ |  | 0.1 | 0.1-1.5 |  | 0.3 | 0.1-4.4 |  | 0.406 | 0.737 |
| f__Peptostreptococcaceae;g__;s__ |  | 0.2 | 0-3 |  | 0.2 | 0.1-7.7 |  | 0.406 | 0.737 |
| g__Clostridium;Other |  | 0 | 0-0 |  | 0 | 0-0 |  | 0.389 | 0.737 |
| g__Bifidobacterium;Other |  | 0.1 | 0-14.9 |  | 0.1 | 0-2.8 |  | 0.427 | 0.75 |
| Collinsella stercoris |  | 2.1 | 0-8.2 |  | 1 | 0-8.4 |  | 0.45 | 0.75 |
| Clostridium neonatale |  | 0 | 0-0 |  | 0 | 0-0 |  | 0.454 | 0.75 |
| g__Sarcina;s__ |  | 0 | 0-0 |  | 0 | 0-0 |  | 0.436 | 0.75 |
| f__Peptostreptococcaceae;Other;Other |  | 0.2 | 0-4.9 |  | 0.5 | 0-8.2 |  | 0.427 | 0.75 |
| f__Fusobacteriaceae;Other;Other |  | 0.1 | 0-0.3 |  | 0 | 0-0.6 |  | 0.45 | 0.75 |
| f__Lactobacillaceae;g__;s__ |  | 0 | 0-0 |  | 0 | 0-0 |  | 0.471 | 0.755 |
| g__Clostridium;s__ |  | 0 | 0-0.3 |  | 0 | 0-1.5 |  | 0.47 | 0.755 |
| g__SMB53;s__ |  | 0.1 | 0-0.2 |  | 0.1 | 0-0.1 |  | 0.496 | 0.772 |
| g__Dorea;s__ |  | 1.7 | 0.7-15.7 |  | 3.7 | 0.5-11.1 |  | 0.496 | 0.772 |
| g__Clostridium;Other |  | 0.1 | 0-0.1 |  | 0 | 0-52.8 |  | 0.545 | 0.825 |
| g__Megasphaera;s__ |  | 0 | 0-0.6 |  | 0 | 0-0 |  | 0.545 | 0.825 |
| f__Rikenellaceae;g__;s__ |  | 0 | 0-0 |  | 0 | 0-0 |  | 0.559 | 0.826 |
| Lactobacillus agilis |  | 0 | 0-0 |  | 0 | 0-0 |  | 0.561 | 0.826 |
| g__Methanobrevibacter;s__ |  | 0 | 0-0.1 |  | 0 | 0-0 |  | 0.583 | 0.847 |
| f__Succinivibrionaceae;g__;s__ |  | 0 | 0-0 |  | 0 | 0-1.6 |  | 0.611 | 0.876 |
| [Eubacterium] dolichum |  | 0 | 0-0.4 |  | 0 | 0-0.3 |  | 0.623 | 0.882 |
| g__Collinsella;s__ |  | 0 | 0-0 |  | 0 | 0-0.2 |  | 0.648 | 0.886 |
| g__Enterococcus;s__ |  | 0.1 | 0.1-4.1 |  | 0.1 | 0.1-1.5 |  | 0.65 | 0.886 |
| g__Fusobacterium;s__ |  | 3.4 | 0.2-9.6 |  | 1.7 | 0.1-36.6 |  | 0.65 | 0.886 |
| g__Parabacteroides;s__ |  | 0 | 0-0.6 |  | 0 | 0-0 |  | 0.701 | 0.938 |
| g__Blautia;s__ |  | 5.1 | 1.4-29.7 |  | 8 | 0.5-19.9 |  | 0.706 | 0.938 |
| Parabacteroides distasonis |  | 0 | 0-0.6 |  | 0 | 0-0.2 |  | 0.731 | 0.95 |
| o__Lactobacillales;Other;Other;Other |  | 0 | 0-0 |  | 0 | 0-0 |  | 0.732 | 0.95 |
| g__Gemella;s__ |  | 0 | 0-0 |  | 0 | 0-0 |  | 0.745 | 0.956 |
| g__Roseburia;s__ |  | 0 | 0-0 |  | 0 | 0-0 |  | 0.759 | 0.962 |
| g__Enterococcus;Other |  | 0 | 0-0 |  | 0 | 0-0 |  | 0.776 | 0.964 |
| g__Candidatus Arthromitus;s__ |  | 0 | 0-0.1 |  | 0 | 0-2.1 |  | 0.778 | 0.964 |
| g__Ruminococcus;s__ |  | 0 | 0-0.1 |  | 0 | 0-0.1 |  | 0.791 | 0.969 |
| Bacteroides ovatus |  | 0 | 0-0.1 |  | 0 | 0-22.4 |  | 0.82 | 0.983 |
| [Ruminococcus] gnavus |  | 3.4 | 0.1-10.5 |  | 3.5 | 0.2-6.9 |  | 0.821 | 0.983 |
| Bacteroides fragilis |  | 0 | 0-0 |  | 0 | 0-0 |  | 0.848 | 0.984 |
| f__Enterococcaceae;Other;Other |  | 0 | 0-0 |  | 0 | 0-0 |  | 0.843 | 0.984 |
| Enterococcus casseliflavus |  | 0 | 0-0 |  | 0 | 0-0 |  | 0.847 | 0.984 |
| Peptostreptococcus anaerobius |  | 0 | 0-0 |  | 0 | 0-0 |  | 0.876 | 0.987 |
| g__Phascolarctobacterium;s__ |  | 0 | 0-0.3 |  | 0 | 0-0 |  | 0.878 | 0.987 |
| Cetobacterium somerae |  | 0 | 0-12 |  | 0 | 0-0 |  | 0.876 | 0.987 |
| g__Actinomyces;s__ |  | 0 | 0-0 |  | 0 | 0-0.1 |  | 0.94 | 0.988 |
| Actinomyces europaeus |  | 0 | 0-0 |  | 0 | 0-0 |  | 0.968 | 0.988 |
| Lactobacillus mucosae |  | 0 | 0-0 |  | 0 | 0-0.1 |  | 0.968 | 0.988 |
| Lactobacillus ruminis |  | 0 | 0-0 |  | 0 | 0-0 |  | 0.969 | 0.988 |
| g__Turicibacter;s__ |  | 0.1 | 0-15.8 |  | 0.2 | 0.1-2 |  | 0.97 | 0.988 |
| g__Butyrivibrio;s__ |  | 0 | 0-0 |  | 0 | 0-0 |  | 0.967 | 0.988 |
| g__Epulopiscium;s__ |  | 0 | 0-1.5 |  | 0 | 0-0.1 |  | 0.97 | 0.988 |
| f__Ruminococcaceae;Other;Other |  | 0 | 0-0 |  | 0 | 0-0 |  | 0.935 | 0.988 |
| g__Anaerobiospirillum;s__ |  | 0 | 0-0.1 |  | 0 | 0-0 |  | 0.908 | 0.988 |
| Akkermansia muciniphila |  | 0 | 0-0 |  | 0 | 0-0 |  | 0.936 | 0.988 |
| g__Corynebacterium;s__ |  | 0 | 0-0.1 |  | 0 | 0-0 |  | 1 | 1 |
| Blautia producta |  | 6.3 | 0.5-17.5 |  | 7.5 | 0.9-22.6 |  | 1 | 1 |
